# Experimental dataset on the characterization of a new photo-calorespirometry setup

**DOI:** 10.1016/j.dib.2025.112157

**Published:** 2025-10-10

**Authors:** Tilman Linke, Sven Paufler, Christian Dusny, Thomas Maskow, Andreas Schmid

**Affiliations:** Helmholtz Center for Environmental Research (UFZ) Leipzig, Permoserstraße 15, D-04318, Germany

**Keywords:** Photo-calorimetry, Light adjustment/quantification, Cyanobacterial phenotypes, Dead cell preparation, Pigment analysis, Morphological characterization, Thermal profiling

## Abstract

This dataset complements the main article “Direct Real-Time Determination of Photosynthetic Efficiency by Photo-Calorespirometry Using Synechocystis sp. PCC 6803 as Model Cyanobacterium” by providing detailed experimental data, calibration procedures, and validation measurements for the photo-calorespirometry (Photo-CR) method. The data ensure precise quantification of light energy input ($P_{I}$), thermal signals, and photosynthetic performance, and validate control systems essential for accurate heat-based assessment of photosynthesis in phototrophic microorganisms.

Light delivery was optimized and in a dual-ampoule calorimetric setup trough calibrated LED–light guide assemblies. including distance-dependent attenuation profiles, peak $P_{I}$ determinations (20 mW), and corrections for thermal asymmetry. A photosynthetically inactive reference system was established by formaldehyde fixation of Synechocystis cells, preserving morphology and pigment content. Physiological and optical validations included Coulter counter cell-size analysis, pigment quantification, and absorption spectroscopy to confirm spectral similarity between fixed and living cells.

Control datasets include thermal profiles of living versus dead cells, medium-only baselines, and light-independent heat generation. Additional measurements capture temporal variations in photosynthetic performance during multi-step light ramp experiments, normalized to biomass via Monod-based growth modeling. Furthermore, a correlation dataset from 18 independent samples links cell number, optical density (OD₇₅₀), and cell dry weight, enabling conversion between these measures.

Collectively, these datasets provide a comprehensive reference for calibrating and validating Photo-CR experiments, supporting reproducible measurement of photosynthetic energy conversion in cyanobacteria. They are intended for reuse in methodological replication, adaptation to other phototrophic microorganisms, optimization of light-dependent calorimetric studies, and the development of standardized reference controls for heat-based photosynthetic efficiency assessment.

Specifications Table

**Table:** 

Subject	Biology
Specific subject area	This work assesses biocalorimetric investigations on photosynthetic energy conversions in aquatic phototrophic microorganisms for academic research and improvements of biotechnological production applications. This includes the design, engineering, and employment of the photo-calorespirometry method using the model cyanobacterium *Synechocystis* sp. PCC 6803, as well as the development of a suitable and stable dead cell reference system.
Type of data	Table, Image, Chart, Graph, Figure, Illustration etc. Raw, Analyzed, Filtered, Processed etc.
Data collection	The Photo-CR system was based on a modified isothermal microcalorimeter (2247 Thermal Activity Monitor, Thermometric AB, Sweden) with one channel integrated into a thermally insulated container on a shaker (Unitwist 400, LTF Labortechnik, Germany), connected to a thermostat (Lauda EcoLine RE204, Lauda GmbH, Germany. Illumination was provided by a LED with driver (Thorlabs Inc., USA) *via* light guides (Volpi, Switzerland). Oxygen optodes monitored O₂ in liquid/gas phases. Cultures of Synechocystis sp. PCC 6803 (Pasteur Collection) were grown in incubators (CLF Plant Climatics, Germany; Infors HT Minitron, Switzerland) and analyzed by spectrophotometer (Biochrom Libra S11, UK), coulter counter (Beckman Coulter, Germany), centrifuge (Heraeus Fresco 17, Thermo Scientific, Germany), and spectrophotometer (Genesys 150, Thermo Scientific, Germany). Light input was calibrated with light meter (Walz ULM-500 with US SQS/L, Walz Group, Germany).
Data source location	*The data was collected at the Helmholtz Center for Environmental Research (UFZ) in Leipzig and is stored in the archives of the UFZ.*
Data accessibility	Linke, Tilman (2025), “Experimental Dataset on the Characterization of a new Photo-Calorespirometry Setup”, Mendeley Data, V2, doi:10.17632/56s83cx3mp.2 [1] Repository name: Experimental Dataset on the Characterization of a new Photo-Calorespirometry Setup Data identification number: doi:10.17632/56s83cx3mp.1 Direct URL to data: https://data.mendeley.com/datasets/56s83cx3mp/1
Related research article	Tilman Linke, Sven Paufler, Christian Dusny, Thomas Maskow, Andreas Schmid. (2025). Direct Real-Time Determination of Photosynthetic Efficiency by Photo-Calorespirometry Using Synechocystis sp. PCC 6803 as Model Cyanobacterium. [2]

## 1 Value of the Data

• The provided data is especially beneficial for researchers in the field of photosynthetic energy conversion studies, biotechnologists and bioprocess engineers, as well as calorimetric method and instrument designers
• **Reference data for system calibration in photo-calorespirometry**
  Includes detailed light calibration procedures from a dual-ampoule calorimetric system. Supports reproducible alignment of symmetrical light input for accurate and reliable energy quantification in phototrophic experiments.
• **Replication and standardization of dead cell reference systems**
  Documents preparation of formaldehyde-fixed Synechocystis cells with physiological and morphological validation, enabling standardized non-photosynthetic-controls in calorimetric studies.
• **Benchmarking of thermal background signals**
  Provides control calorimetric profiles for medium, dead cells, and dark conditions, helping researchers to distinguish baseline heat contributions from true photosynthetic signal.
• **Pigment-level normalization of photosynthetic activity**
  Supplies spectrophotometric data on chlorophyll and carotenoid content in living and fixed cells, facilitating normalization of energy conversion rates to pigment content and enabling cross-study comparisons.
• **Linking optical density, biomass, and cell counts**
  Correlation data between OD₇₅₀, dry cell mass, and cell number allow estimation of cell densities and biomass yields, supporting kinetic modeling and growth predictions.
• **Time-resolved photosynthetic response data**
  Provides activity data across from multistep ramp experiments, enabling dynamic modeling of light-response behavior in cyanobacteria and informing light-regulated process designs.

## 2 Background

Photosynthetic efficiency (*PE*) in phototrophic organisms is crucial for sustainable bioresource production, yet remains difficult to quantify comprehensively due to fluctuating environmental conditions and limitations of existing measurement techniques. This study aimed to develop and validate a novel method—photo-calorespirometry (Photo-CR)—for real-time, non-invasive quantification of photosynthetically fixed energy and oxygen evolution in phototrophs. This includes the symmetrical adjustment and determination of light energy inputs, as well as the development and verification of the dead cell reference system to solely derive heat data that can be assigned to the actual photosynthetic process.

## 3 Data Description

### 3.1 Optimization of light input and distribution in a dual-ampoule calorimetric system

Accurate determination of the light energy input (*P_I_*) is essential for quantifying photosynthetic efficiency (*PE*) and balancing light distribution between the measurement (M) and the reference side (R) side. The methodology for determining *P_I_* (see the main article) used a custom-designed ampoule with a bottom opening to measure *P_I_* (repository data). Increasing the distance between the LED output and the ampoule reduced internal light intensity, following a non-linear attenuation due to reflective losses. The relationship was described as $\Phi \;\left(d\right)=\Phi \left(d=0\right)+B\cdot \left(e^{\left(C\cdot D\right)}-1\right)$, where B and C are constants. This equation was fitted with OriginPro.

Symmetrical light input was achieved by adjusting light guide position relative to the LED, yielding a peak of *P_I_* of approximately 20 mW (repository data). Measurements were performed outside the calorimetric vessel and without liquid which may have introduced minor inaccuracies. Displacements or misalignments of the light guides caused slight offset, observed as asymmetry in medium vs. medium controls (repository data). Stability in *P*_I_ was also affected by guide movement or wear. Regular monitoring with a light sensor and medium only controls is therefore recommended. To improve reproducibility, shorter and/or newer light guides are suggested.

### 3.2 Establishing a photosynthetically inactive reference system

For Photo-CR, both sides must receive identical light energy input and conditions. The M-side contained living cells, while the R-side contained photosynthetically inactive but morphologically intact cells. Formaldehyde fixation was selected as the most suitable method eliminating photosynthetic activity (confirmed as absence of colony-forming units) while preserving morphology. Coulter counter analysis and microscopy confirmed that fixation did not alter cell size and morphology (repository data). Calorimetric controls in darkness showed negligible heat release from dead cells, indication minimal lysis and degradation during the measurement (repository data).

### 3.3 Physiological and optical validation of the dead cell controls

Absorption spectra and pigment content were measured to validate the similarity between living and fixed cells. Chlorophyll ∝ and carotenoid contents were nearly identical in both cases (living: 2.7 ± 0.7 % chlorophyll ∝ and 1.6 ± 0.3 % carotenoids; fixed: 2.6 ± 0.5 % chlorophyll ∝ and 1.3 ± 0.3 %, respectively) (repository data), Absorption scans also overlapped, with maxima at 430–440 nm, 620–640 nm, and 660–680 nm (repository data). These results confirm that fixation preserved light-harvesting pigments and spectral properties relevant for Photo-CR experiments.

### 3.4 Thermal profiling and calorimetric offset corrections

The initial development steps of this system were described in [3], and subsequent work advanced it into a photo-calorespirometer through the integration of oxygen optodes [4]. Following further refinement, the setup was optimized for cyanobacterial studies by employing a monochromatic red-light LED (660 ± 20 nm; Thorlabs Inc., USA). Calorimetric experiments (repository data) compared signals from living cells, fixed cells against medium. Living cells showed the highest endothermic signals, representing total biomass-associated heat fluxes (metabolic heat, photochemical/non-photochemical quenching, and fluorescence). Fixed cells generated lower signals, consistent with the absence of photosynthesis. The difference between living and fixed cells profiles matched the photosynthetic fixed energy (*P_PS_*). This validated the physical assumptions behind the Photo-CR energy balance (see Eq. (1) [2]).

Medium only measurements revealed a small offset (approx. 300 µW), attributed to residual asymmetries in light distribution. This offset was subtracted in data processing. Dead cells in darkness released minimal heat (approx. 20 µW), which was negligible compared to and could be disregarded due to photosynthetic signals.

### 3.5 Photo-Calorespirometric data processing

All data were processed in OriginPro. Calorimetric and oxygen datasets were imported, ramp segments, and gas-phase oxygen data smoothed. Baseline subtraction removed systematic offsets; caused by unequal light conditions, providing *P_PS_*. Using the recorded light energy inputs at each time point, Photosynthetic efficiencies were calculated as *P_PS_*/*P_I_* as a function of light energy input and normalized to biomass estimates obtained via Monod modeling from optical density before/after experiments.

The oxygen production rate in the liquid phase ($r_{O2}$in µmol L^−1^ s^−1^) was obtained from the measured oxygen concentration in the liquid phase (*O_L_* in µmol L⁻¹) and in the gas phase ($\xi_{O}^{V}$ in vol. %) according to:

$$r_{O2}=\frac{dO_{L}}{dt}+k_{V}\cdot \left(O_{L}-k_{H}\cdot \xi_{O}^{V}\cdot \pi \right)$$

The second term of the equation corrects for the oxygen exchange with the gas phase. *k_V_* in s^-1^ is the experimentally determined gas exchange coefficient and $k_{H}=1.4\cdot 10^{-8}\;mol_{O2}\;L^{-1}\;Pa^{-1}$ is the Henry coefficient [5]. π is the atmospheric pressure. The oxycaloric equivalent was obtained as the slope of ***P_PS_*** vs. *r_O2_*.

### 3.6 Correlation between biomass and cell number

To relate photosynthetic performance of a single cell, correlations between cell counts, optical density (OD_750_), and cell dry weight were established. 18 samples with varying OD_750_ were analyzed by Coulter counter, and cell dry weight was calculated using a strain specific correlation factor (0.17 $g_{CDW}\;L^{-1}$ per OD_750_) [6]. Regression analysis yielded conversion factors of 4.25 × 10^7^ $cells\;ml^{-1}$ per OD_750_ and 2.50 × 10^7^ $cells\;ml^{-1}g_{CDW}^{-1}$ (repository data). Strong linearity was observed at low ODs, while higher deviations at OD > 0.4 were likely due to required sample dilutions for Coulter counter operation (repository data).

## 4 Experimental Design, Materials and Methods

### 4.1 Methods

#### 4.1.1 Photo-Calorespirometry setup

The Photo-CR system was built from a modified isothermal microcalorimeter (TAM 2247, Thermometric AB, Sweden). One calorimetric channel was relocated into a thermally insulated container mounted on a shaker (Unitwist 400, LTF Labortechnik, Germany) to reduce self-shading. The container was thermostated at 30 ± 0.2 °C (Lauda EcoLine RE204, Germany). Illumination was provided by an LED (Thorlabs, USA) coupled to two light guides (Volpi, Switzerland) directed onto the lids of the measurement (M) and reference (R) ampoules. Each ampoule was placed on Peltier elements, and heat flux was quantified via the Seebeck effect and recorded with Digitam software following calibration. Aluminum rings improved thermal contact and reduced pre-experimental stabilization time. In addition, the M-side was equipped with oxygen optodes in both the liquid phase and headspace to monitor O₂ dynamics and account for gas–liquid transfer.

#### 4.1.2 Cultivation and sample preparation

*Synechocystis* sp. PCC 6803 (Pasteur Culture Collection, Paris) was maintained on BG11 agar at 30 °C under white light (approx. 50 µmol photons m⁻² s⁻¹, 58 % RH). Colonies were transferred weekly to 10 mL YBG11, then subcultured every three days into 24 mL fresh medium. For acclimation, liquid cultures were grown at 30 °C, 75 % RH, 150 rpm, under red light (660 ± 20 nm, approx. 50 µmol photons m⁻² s⁻¹). For calorimetric measurements, cultures were adjusted to OD₇₅₀ = 0.05 in 10 mL medium. The R-side was filled with 10 mL formaldehyde-killed cells, prepared using methanol-free paraformaldehyde and verified as non-viable on BG11 plates. All measurements used cultures from the same wild-type stock, with biological replicates grown on separate days at identical growth stages.

#### 4.1.3 Determination of intracellular chlorophyll α content and carotenoids

On measurement days, 1 mL samples of live and dead cultures were centrifuged (15,000 g, 7 min, 4 °C), extracted with cold methanol (−18 °C, 20 min, dark), and re-centrifuged. Absorbance of the supernatant was measured at 665, 470, and 750 nm using methanol as blank. Chlorophyll α and carotenoid contents were calculated according to [7,8] and normalized to biomass, using OD₇₅₀ and a dry weight correlation factor of 0.17 g L⁻¹ [6].

#### 4.1.4 Calorimeter settings and measurement protocol

The TAM was calibrated before each run using a built-in 3000 µW resistor. Ampoules connected to light guides were inserted stepwise into the calorimeter to allow thermal stabilization. Measurements were conducted at 30 °C with horizontal shaking (124 rpm) under red LED light (660 ± 20 nm). Each run included five light ramps (0 → 100 mA over 3600 s), a plateau (100 mA, 1800 s), and return to 0 mA, interspaced with 1800 s dark intervals. After each run, OD₇₅₀ and Coulter counter analyses were performed to verify biomass growth and exclude contamination; contaminated samples were discarded. Data processing were performed in OriginPro.

#### 4.1.5 Determination of light energy input

Prior to experiments, light input for M- and R-sides was measured using a custom ampoule with a Walz ULM-500 light meter. Radiation flux (Φ) was measured at four distances (3–53 mm range) from the LED at 100 mA with three replicates per distance. The averaged data were used to create an interpolation function in OriginPro. Experimental runs used 660 ± 20 nm red light at calibrated distances.

## Limitations

The described data in this article is limited to application with the wildtype *Synechocystis* sp. PCC 6803 strain under monochromatic red-light (660 ± 20 nm). The derived physiological parameters in this study refer solely to the respective experimental conditions*.*

## Ethics Statement

We declare that this work did not include any human or animal testing without data collection from social media platforms.

## Credit Author Statement

**Tilman Linke:** Conceptualization, Methodology, Data Acquisition and Processing, Writing, Visualization. **Sven Paufler:** Methodology, Software. **Christian Dusny:** Conceptualization, Supervision, Reviewing and Editing. **Thomas Maskow:** Conceptualization, Supervision, Writing, Reviewing and Editing, Data Interpretation. **Andreas Schmid:** Conceptualization, Supervision, Writing, Reviewing and Editing.

## Data Availability

Mendeley DataExperimental Dataset on the Characterization of a new Photo-Calorespirometry Setup (Original data).
